# Cholera Outbreak in Senegal in 2005: Was Climate a Factor?

**DOI:** 10.1371/journal.pone.0044577

**Published:** 2012-08-31

**Authors:** Guillaume Constantin de Magny, Wassila Thiaw, Vadlamani Kumar, Noël M. Manga, Bernard M. Diop, Lamine Gueye, Mamina Kamara, Benjamin Roche, Raghu Murtugudde, Rita R. Colwell

**Affiliations:** 1 MIVEGEC, UMR 5290 CNRS-IRD-UM1&2, Centre de recherche IRD, Montpellier, France; 2 National Centers for Environmental Predictions, Climate Prediction Center, Camp Springs, Maryland, United States of America; 3 Clinique des Maladies Infectieuses du Centre National et Universitaire de Fann, BP 5035, Dakar Fann, Sénégal; 4 Unite Mixte International de Recherche - Environnement, santé, sociétés (UMI 3189), Universite Cheikh Anta Diop de Dakar (UCAD), BP 5005, Dakar Fann, Sénégal; 5 Direction de la Météorologie Nationale, Agence Nationale de la Météorologie Sénégal, Aéroport Léopold Sédar SENGHOR, BP 8257, Dakar Yoff, Sénégal; 6 UMI IRD/UPMC 209 UMMISCO, Centre IRD de Bondy, Bondy, France; 7 Earth System Science Interdisciplinary Center, University of Maryland Research Park (M-Square), College Park, Maryland, United States of America; 8 University of Maryland Institute for Advanced Computer Studies, College Park, Maryland, United States of America; 9 Maryland Pathogen Research Institute, University of Maryland, College Park, Maryland, United States of America; 10 Department of Environmental Health, Johns Hopkins Bloomberg School of Public Health, Baltimore, Maryland, United States of America; University of Swansea, United Kingdom

## Abstract

Cholera is an acute diarrheal illness caused by *Vibrio cholerae* and occurs as widespread epidemics in Africa. In 2005, there were 31,719 cholera cases, with 458 deaths in the Republic of Senegal. We retrospectively investigated the climate origin of the devastating floods in mid-August 2005, in the Dakar Region of Senegal and the subsequent outbreak of cholera along with the pattern of cholera outbreaks in three other regions of that country. We compared rainfall patterns between 2002 and 2005 and the relationship between the sea surface temperature (SST) gradient in the tropical Atlantic Ocean and precipitation over Senegal for 2005. Results showed a specific pattern of rainfall throughout the Dakar region during August, 2005, and the associated rainfall anomaly coincided with an exacerbation of the cholera epidemic. Comparison of rainfall and epidemiological patterns revealed that the temporal dynamics of precipitation, which was abrupt and heavy, was presumably the determining factor. Analysis of the SST gradient showed that the Atlantic Ocean SST variability in 2005 differed from that of 2002 to 2004, a result of a prominent Atlantic meridional mode. The influence of this intense precipitation on cholera transmission over a densely populated and crowded region was detectable for both Dakar and Thiès, Senegal. Thus, high resolution rainfall forecasts at subseasonal time scales should provide a way forward for an early warning system in Africa for cholera and, thereby, trigger epidemic preparedness. Clearly, attention must be paid to both natural and human induced environmental factors to devise appropriate action to prevent cholera and other waterborne disease epidemics in the region.

## Introduction

Cholera is an acute diarrheal illness caused by toxigenic *Vibrio cholerae* and occurs as widespread epidemics that are a major public health concern for Africa. The role of climate in cholera transmission has been extensively investigated in recent years because of growing concern about the effects of climate change on infectious disease dynamics [1], [2], [3]. Lipp *et al.* (2002) and Pascual *et al.* (2002) reviewed the effects of climate on cholera transmission, mainly in Asia, and in particular, the effect of rainfall. It is well known that cholera affects primarily large populations with little or no access to safe water and proper sanitation. Weather and climate play a critical role because of their effect on water quality [4], [5], [6], and can also affect water quantity, i.e. water availability. It has now been well established that environmental conditions combined with climate are important factors in the dynamics of cholera, influencing the abundance and ecology of the pathogen naturally present in the environment and increasing exposure and risk of human infection. This pattern is well documented, especially for Southeast Asia, but only a limited number of studies have focused on Africa, currently one of the most impacted continents [7], [8], [9], [10], [11].

**Figure 1:**
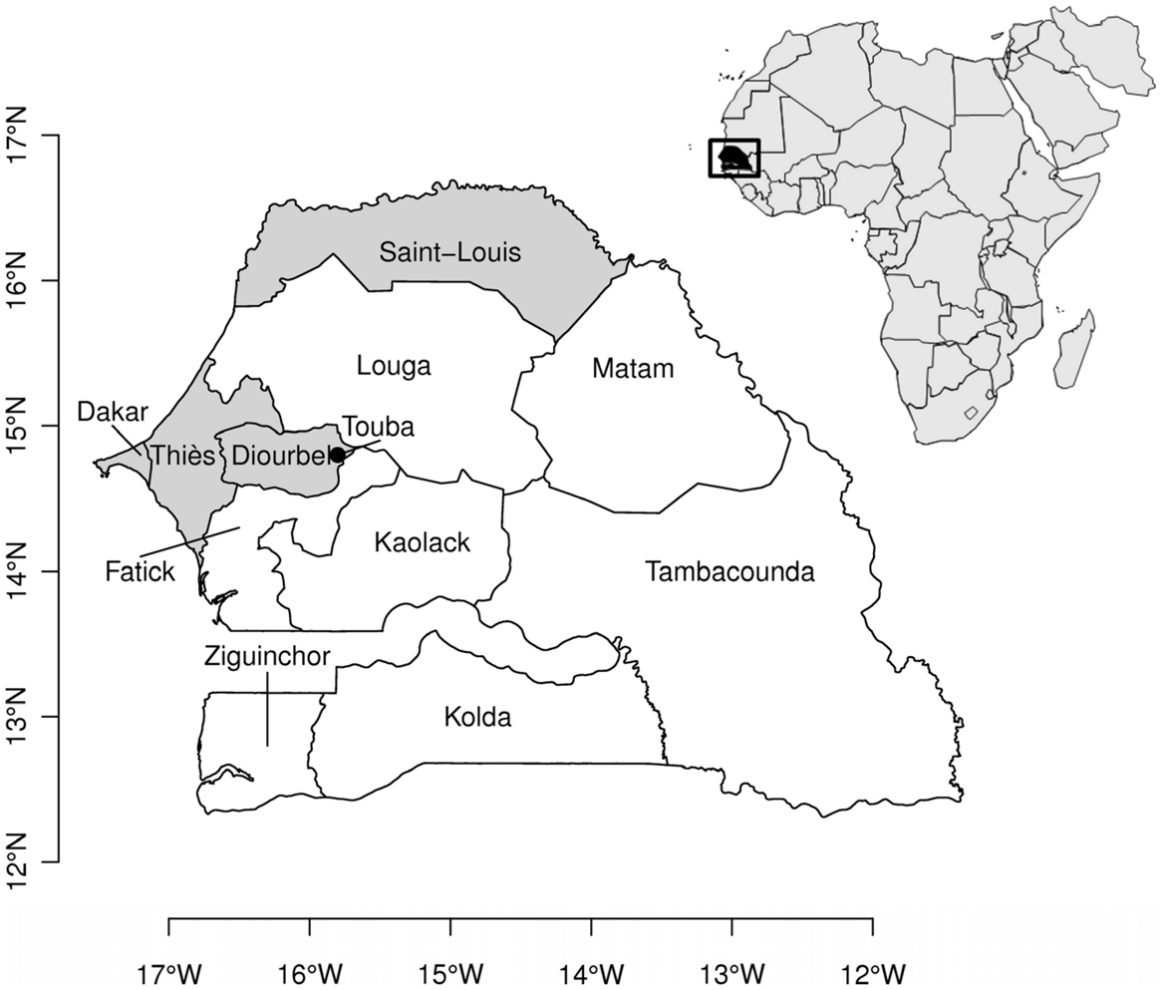
Map of Republic of Senegal and its regions.

**Figure 2:**
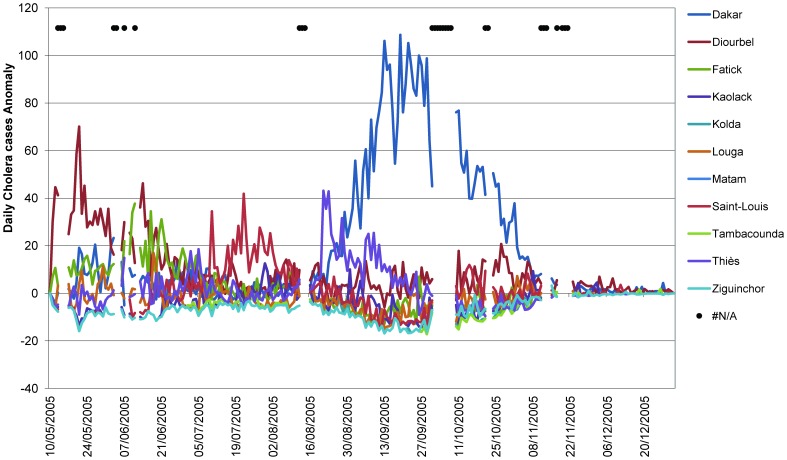
Temporal variability of cholera in Senegal. Daily accumulation of cholera cases anomaly observed over 11 regions of Senegal between May 10, and December 31, 2005.

The Republic of Senegal, located south of the Senegal River in Western Africa and bordering the Atlantic Ocean to the west (Figure 1), has experienced several severe episodes of cholera since the first cases were reported in 1971 [12]. In 2004-2005, as part of a significant series of cholera outbreaks in West Africa [12], an epidemic took place in Senegal, resulting in 31,719 cases, i.e., 293 cases/100,000 habitants, with 458 deaths (case fatality rate (CFR) of 1.44%). This epidemic was the largest recorded by the World Health Organization (WHO) for Africa that time [13], and the most severe of those recorded for that country since 1970 [12], as it represents 46.9% of the total morbidity accumulated over 41 years (1970-2010, Table S1). Among the bordering countries of Senegal in 2005, then Guinea-Bissau was the most affected by cholera with 25,111 cholera cases, and Mauritania, Guinea, Mali and Gambia with 4132, 3821, 1178, and 214 cholera cases, respectively. From January to April 2005, districts in the Diourbel region to the east of Dakar were the most severely affected by cholera, and cases recorded in the Touba District where the Mouride pilgrimage takes place annually in the holy city of Touba (Figure 2) accounted for the large increase in the number of cases [12]. A resurgence of cholera occurred in Senegal after August 15, 2005, mainly in the region around Dakar, following the devastating floods that affected the western sector. Seasonal rainfall in Senegal is associated predominantly with the northward migration of the continental Intertropical Convergence Zone (ITCZ) during the boreal summer months, with the amount of rainfall decreasing from the south of the country to the north and increasing from June to about August and tapering off into early fall [14]. Rainfall over Senegal is known to be influenced by both the Atlantic, through surface temperature gradients between the Gulf of Guinea, the tropical north Atlantic, and continental West Africa, such that a warmer than normal tropical north Atlantic tends to favor enhanced precipitation in this region. The Pacific SST and the Indian Ocean are also important, primarily related to the El Nino-Southern Oscillation (ENSO). Hence, Senegal, as in many regions in the tropics can be sensitive to global climate modes and climate change signals [14], [15].

Availability of daily notification of cholera cases along 11 regions of Senegal between May 10, 2005, and December, 31, 2005, offered an ideal context for dissecting patterns of cholera dynamics and rainfall. In this study, a retrospective investigation of the rainfall pattern that occurred during the resurgence of cholera in the Dakar Region in the second half of the year 2005, provided a quantitative illustration of climate context and dynamics of cholera transmission. We also studied patterns of cholera and rainfall in three other regions of Senegal, including Diourbel and Thiès, which lie in the interior to the east of Dakar, as well as Saint-Louis, located near the northern coast (Figure 1). At the country scale, we compared the rainfall pattern for Senegal in 2005 to patterns of rainfall observed between 2002 and 2004 to characterize possible differences. Finally at the ocean basin-scale, we investigated the pattern of the SST gradient in the tropical Atlantic Ocean between 2002 and 2005, since the Atlantic SST primarily influences West African rainfall and that of Senegal, in particular.

## Methods

### Epidemiological Data

The daily record of new cholera cases over the 11 regions of Senegal between May 10, 2005, and December 31, 2005, was obtained from the Senegal Ministry of Health website in 2006 (www.sante.gouv.sn). The WHO definition was used to define cholera [16], and cholera cases were confirmed by culture at the beginning and end of the outbreak. To limit potential bias of population size in the different regions selected for comparison, the data were converted to incidence per 100,000 per day. Region specific population sizes in 2005 were retrieved from the website of the Senegal National Agency of Statistics and Demography (http://www.ansd.sn/). Missing data in the epidemiological dataset representing 27 days (11.4%) of the 236 days between May 10 and December 31, 2005 (Figure 2) were determined by the nearest neighbor interpolation as a daily moving average of a 7-day window prior to the missing data. We computed cholera case anomaly by subtracting the mean over all the data to each data unit, which is equivalent to centering the data.

### Climate Data

Daily precipitation was extracted from satellite rainfall estimates provided by the Climate Prediction Center (CPC) at 10 km resolution [17]. The daily rainfall anomaly was obtained by subtracting from the rainfall value for a given day, mean of the rainfall value of the same day observed over four years. We then accumulated the anomaly year by year. The country mean rainfall accumulated anomaly is presented in Figure S1. Spatial patterns of heavy rainfall events over Senegal between 2002 and 2005 were illustrated by maps of the annual mean of accumulated rainfall greater than 200 mm.day^−1^, computed by a 7-day average moving window. SST data were obtained from the NCEP Climate Data Assimilation System (CDAS) [18]. The SST gradient in the Atlantic Ocean corresponded to the difference between SST in a 5-degree box centered at 15^o^N-20^o^W (SST_North_) and a 10-degree box centered on the equator at 0^o^ longitude (SST_South_). All climate data were retrieved for the time period between January, 2002, and December, 2005. The SST anomaly was similarly obtained as the rainfall anomaly.

### Statistical Analysis

To quantify the link between rainfall and cholera morbidity in 2005 over Dakar region, we have applied a cross-correlation analysis between the two time series [19].

## Results

The total number of new cholera cases for the 11 regions of Senegal during the time period under consideration showed three dynamical regimes (Figure 2). The first was between May 10 and July 5, 2005. The total number of daily cholera cases ranged between 64 and 175, with the maximum occurring on May 20 in Diourbel, Fatick, and Dakar. After a first transition on July 6, 2005, the total number of new cases decreased and oscillated between 42 and 105 until August 14, 2005, with the maximum on July 9, 2005, recorded in the region of Saint-Louis. The third dynamical regime began on August 15 and continued until the end of the year, corresponding to cholera resurgence, with a rapid and steady increase in number of cases, reaching 120 new cases per day between September 10 and October 19, 2005, until the outbreak ended in early November, 2005. The most affected regions during this sharp increase in cholera were Dakar and Thiès with the total number of cholera cases in Senegal during this period of 15,798 and Dakar registering 5,474 cases (34.6%), an increase of approximately 50% of the total number of cholera cases (31,719 cases) recorded in Senegal during 2005 [12].

**Figure 3:**
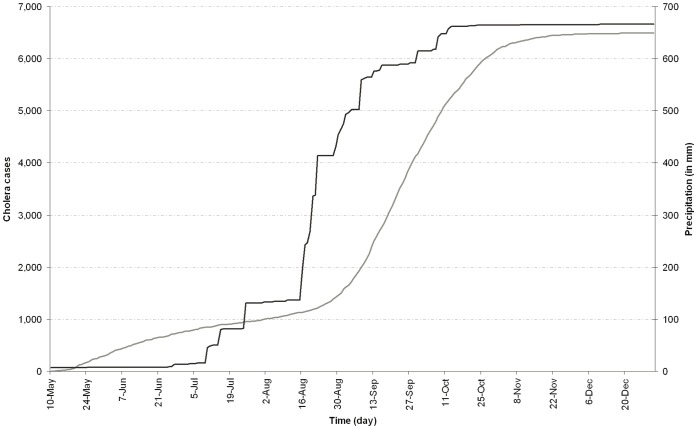
Patterns of daily accumulated number of cholera cases and rainfall for the Dakar region. Cholera cases and rainfall between May 10, and December 31, 2005, are represented by grey and black lines, respectively.

Figure 3 shows the accumulated number of daily cholera cases and rainfall in the Dakar region. A sudden, intense rainfall event measured by satellite, began August 15, 2005, and lasted approximately 7 days, with a total of 277 mm accumulated precipitation. Before this event occurred, the accumulated precipitation from the beginning of the year was 137 mm, hence the intensity of this episode. There was a short pause following this event before the rains resumed, but with less intensity, persisting through September 16, 2005. Accumulated annual total precipitation by this date was 587 mm. Precipitation events thereafter were scattered and the rainy season ended by October 13, 2005, with the annual total of 677 mm for Dakar.

From May 10 to August 15, 2005, a total of 1,124 cases of cholera were recorded for Dakar. On August 22, 2005, seven days after the heavy precipitation had begun, the number of new cases increased significantly from 15 per day to a maximum of approximately 123 new cases per day, 28 days later, on September 12. Our statistical cross-correlation analysis emphasizes that dynamics of cholera cases and rainfall are linked strongly with a lag of 23 days (Figure S2). The number of new cases that occurred daily remained more than or equal to 90 until the beginning of October, 2005, and then decreased. The epidemic declined at the end of the year, when 10 or fewer new cases per day were recorded (November 14, 2005) and the last of the new cases was reported on December 26, 2005. The incidence rate of cholera in Dakar over this period was 224 cases/100,000 inhabitants.

**Figure 4:**
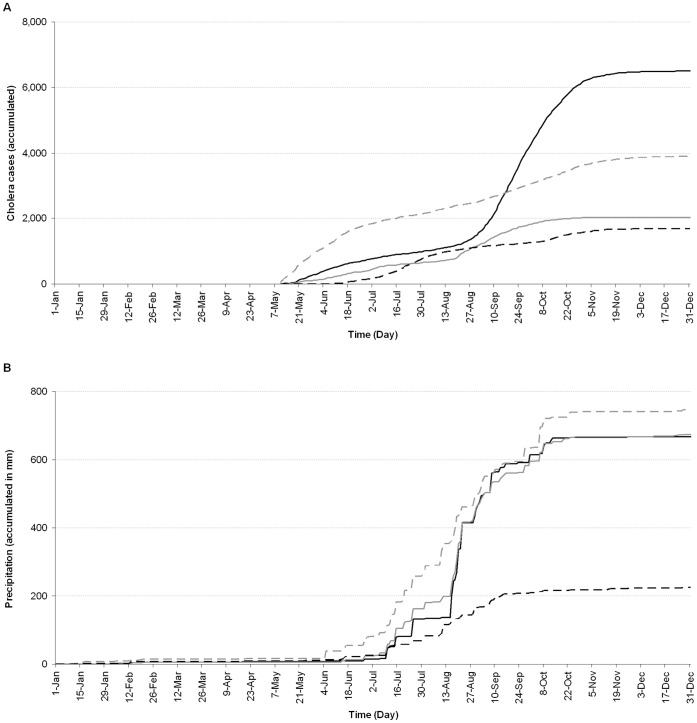
Patterns of daily accumulated cholera incidence (A) and rainfall (B) for four regions of Senegal in 2005. Patterns for regions of Dakar, Thiès, Saint-Louis, and Diourbel are represented by solid black, solid grey, dashed black and dashed grey lines, respectively.

A comparison of accumulated incidence of cholera and total rainfall for Dakar, Thiès (55 km eastward), Diourbel (130 km eastward), and Saint-Louis (180 km northeastward) revealed contrasting patterns (Figure 4). The epidemiological pattern for Dakar and Thiès showed similar trends, with a slightly lower incidence of cholera in Thiès after September 16, 2005 (Figure 4A). On August 20, 2005, a break in the Thiès curve corresponded to a shift in cholera incidence, from 0.5 cases/100,000 per day (Standard Deviation (SD) = 0.39) to 2 cases/100,000 per day (SD = 0.72), on average, for the next 30 days. Comparing the four regions, the epidemic declined, first in the Thiès region, with no new cases recorded after November 2, 2005, with an incidence rate of 146 cases/100,000 inhabitants for the entire period. As stated above, cholera incidence was higher in the Diourbel region between May 10 and July 4, 2005, with a daily average incidence rate of 2.76 cases/100,000 (SD = 1.23) dropping to 0.91 cases/100,000 (SD = 0.37) over the next two months and increasing slightly before decreasing again to an average of 0.21 cases/100,000 (SD = 0.17) on November 14, 2005. Incidence rates decreased thereafter to the end of the year. With respect to population size, the accumulated cholera incidence for the Diourbel region of 321 cases/100,000 inhabitants was greater than for the other three regions including Dakar. Average cholera incidence for Saint-Louis increased significantly on June 8, 2005, to 1.82 cases/100,000 (SD = 1.26) until August 14, 2005, represented by a break in the curve (Figure 4A). The cholera incidence dropped to an average of 0.68 cases/100,000 (SD = 0.37) between August 15 and October 8, 2005, before rising again to an average of 1.44 cases/100,000 (SD = 0.73) up to November 6, 2005, dropping to an average of 0.11 cases/100,000 (SD = 0.16) at the end of the year. Incidence for Saint-Louis, May 10 to December 31, 2005, was 212 cases/100,000.

While accumulated rainfall patterns for Dakar and Thiès were similar with both regions recording essentially the same rainfall totals for 2005 (667 and 673 mm, respectively), the Diourbel and Saint-Louis rainfall patterns, however, were different. In Diourbel, rain began earlier in the season and was evenly distributed from June 4 to October 29, 2005, receiving the highest accumulated precipitation of 745 mm during 2005. In Saint-Louis, the onset was in mid-June and precipitation was frequent until September 15, 2005. Rainfall, thereafter, was scattered and the total accumulated precipitation of 224 mm was the lowest of the four regions during 2005.

**Figure 5:**
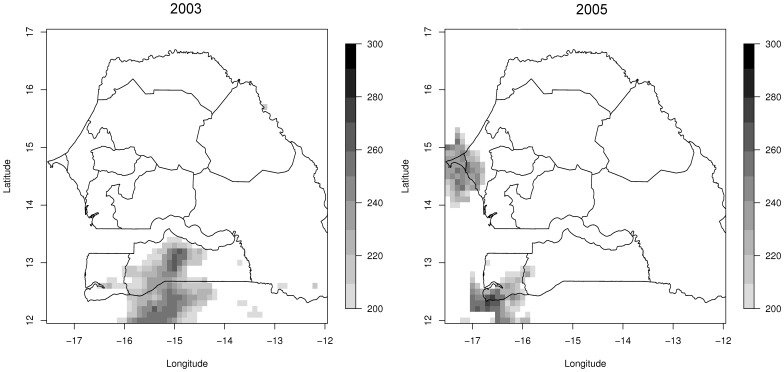
Rainfall accumulation patterns. Spatial distribution over Senegal of annual average rainfall accumulation over 200 mm for a 7 days daily sliding window for years 2003 and 2005. The datasets were screened for erroneous values.

The spatial patterns of heavy precipitation events over Senegal between 2002 and 2005 are illustrated by maps of annual mean accumulated precipitation of greater than 200 mm.day^-1^, computed by a 7-day average moving window (Figure 5). Seven days of accumulated rainfall greater than 200 mm.day^-1^ were observed only in 2003 and 2005. In 2003 it was mostly the Kolda region where the high level of accumulated rainfall was observed. In 2005, Dakar and Thiès along with the south of Zinguichor region also received higher levels of rainfall accumulated over 7 days. Analysis of the temporal profiles of the country mean accumulation of rainfall anomaly showed positive anomalies for these years (Figure S1). A deficit of rain was observed in the first half of 2003, while an excess of rain was observed after the first one third of 2005. In 2002, an excess of rain was accumulated in the first half of the year, when a significant deficit of rain was accumulated to the end of the year. For 2004, almost the entire year was deficient in rainfall.

**Figure 6:**
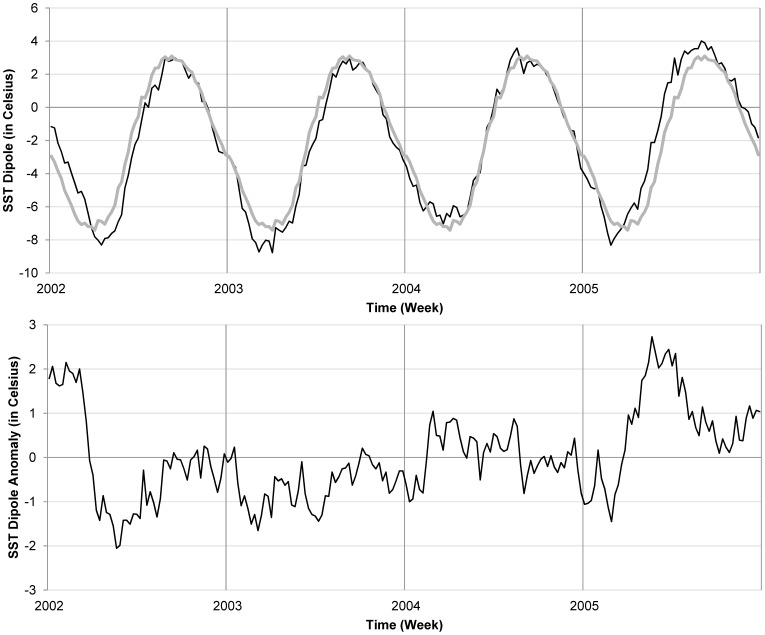
SST gradients comparison. (Top panel) SST dipole time series (black line) between 2002 and 2005 and the seasonal dipole mean computed over the four year period (grey line). (Bottom panel) SST dipole anomaly.

At the basin-scale over the Atlantic Ocean, the gradient between SST_North_ and SST_South_ displayed a different trend during 2005, compared with 2002, 2003, and 2004 (Figure 6, top panel). For 2005, the positive gradient (SST_North_ warmer than SST_South_) appeared earlier in the year, on June 12, while during 2004, 2003, and 2002, the positive gradient did not appear until June 29, July 20, and July 13, respectively. In addition, the gradient was at its maximum (+4°C), on August 31, 2005, and that was maximum for the four year period. Furthermore, the SST gradient remained positive for the longest time during 2005, turning negative only after November 23, 2005. The SST dipole anomaly gradient is positive from March to December 2005, the longest period of time compared to 2002, 2003, and 2004 (Figure 6, bottom panel). It should be noted that a positive gradient corresponds to warmer northern tropics which favors a northward position of the Inter Tropical Convergence Zone and thus anomalously high rainfall in the region under consideration in this study (see [20] for the low-frequency variability gradient). The mechanisms responsible for this variability are beyond the scope of this study but useful details related to this study are reported in [21], [22].

## Discussion

Flood events are natural disasters that are frequently associated with increased risk of water- and vector-borne infectious diseases [23]. A major risk factor for water-borne disease outbreaks, such as cholera, is the contamination of drinking water facilities. For populations having to rely on untreated water taken directly from rivers, ponds, or tube wells, and those lacking proper sanitation, extreme events like floods can be especially disastrous [24], [25], [26]. With projections of enhanced extreme events under global warming [27], cholera offers an ideal paradigm for climate change and human health especially when combined with other natural disasters and human interactions as evidenced in Haiti [28]. Results of this study show the heavy rainfall that occurred during the wintering of 2005 in the Dakar region and at the origin of floods exacerbated the cholera epidemic, which had been underway in the eleven regions of Senegal before the floods (Figure 2). Analysis of the precipitation showed Dakar and the surrounding region received a significant amount of rain (277 mm) within a very short period of seven days, in addition to the 137 mm before the floods. For the Thiès region adjacent to Dakar, rainfall during this period was similar to that of Dakar with an accumulated precipitation of 217 mm. It is interesting to note that cholera incidence increased immediately after heavy precipitation in Dakar and Thiès. The dynamic character and significant size of the cholera outbreak in Dakar compared to that of Thiès was most likely related to population size of the region (2,438,154 inhabitants) and density (4,433 inhabitant/km^2^) [29]. The rainfall and epidemiological patterns of Dakar and Thiès, compared with those of the two other regions of Senegal, showed that the total rainfall accumulated for the year was not the determining factor when compared to the Diourbel region (745 mm of rain). Instead, it was mainly the temporal dynamics of the precipitation, namely, sudden and heavy rain, which was likely the driving factor. In Dakar, low-lying neighborhoods and deficient water drainage led to water stagnation, forcing approximately 50,000 persons to be displaced [30] and rendering the region more vulnerable to cholera. This combination of factors was highly conducive to a sudden and massive increase in cholera, i.e., cholera that was already occurring in the population [23], and in the number of the causative agent, *Vibrio cholerae*, already present in the aquatic environment. Epidemiological studies have shown that lack of adequate potable water, effective sanitation, and appropriate primary road drainage to prevent suppression of the natural water catchment, provide optimal conditions for cholera [30].

Interestingly, the incidence of cholera in the Diourbel region was higher than in Dakar during the time of this study. Diourbel has the second highest population density (254 inhabitants/km^2^) in Senegal, after Dakar [31]. The population distribution for this land area is, however, highly uneven with approximately 49% living in Touba, the holy city of Mouridism, an important trade and market place for the region. It is characterized by an intense migration flux with adjacent regions. One hypothesis is that the officially recorded population size of the Diourbel region at the time of the cholera outbreak did not include migrants and temporary inhabitants that suffered from cholera. The result would be underestimation of the population size from which to compute incidence, introducing bias in the calculation. Examination of the data at a finer spatial scale and integration of the estimate of migrants and temporary habitants should provide a more accurate measurement of the incidence of cholera in Diourbel.

The absence of recorded cholera cases in Senegal up to 2004 when cholera was reported suggests Senegal can be considered an epidemic region [12]. This is reinforced by investigating similar climate conditions as occurred in August 2005, in Dakar and the surrounding regions (Figure 5). For example in 2003, in the Kolda region and to the north of Guinea-Bissau there was a concentrated seven day accumulated precipitation of >200 mm without similar cholera epidemiological consequences. Since 2004, Senegal has reported cholera cases every year, with 1,263 cases in 2008 [32], raising serious concerns about cholera being endemic [33]. More recently, heavy rainfall over West Africa, between June and September, 2009, affected a population of 600,000, mainly in Burkina Faso, Senegal, Ghana, and Niger [34]. Cholera cases were not recorded in Senegal during 2009, reinforcing the hypothesis that flooding can amplify cholera transmission within a population.

As cholera cases were defined according to the WHO definition, it may introduce some reporting bias. To alleviate that bias, the first cholera cases caused by *V. cholerae* O1 El Tor and cases at both the beginning and end of the outbreak were confirmed by culture [35]. It should be noted that underreporting of cholera is a recurrent issue, most particularly in the context of very high morbidity and can weaken the reliability of a data set. Because we focused our study on a single outbreak, however, the bias is limited.

Africa is considered to be the most severely affected by cholera outbreaks, exemplified by the 179,257 cases in 2008 that represented 94% of the total number of cholera cases reported worldwide [32]. In addition, if this official number represents only 10% of actual cases in the most optimistic view of the WHO [36], then there could actually have been almost 1,792,570 cases and 50,730 deaths (case fatality rate equal to 2.83%) in 2008 in Africa.

Analysis of the SST gradient between the Gulf of Guinea and an area off the coast of Senegal showed that SST variability in the Atlantic Ocean in 2005 was significantly anomalous compared to that during 2002 to 2004. The positive gradient appeared earlier in the year, reaching a maximum in early September, 2005, which is important since West African rainfall and that of Senegal, in particular, are primarily influenced by the Atlantic SST [14]. During the 2005 rainfall season, the Atlantic dipole mode (ADM), featuring a warm tropical north Atlantic and a cool Gulf of Guinea was prominent [37]. The persistence of enhanced temperature gradients maintained a deep penetration of the monsoon flow farther north into the Sahel and resulted in heavy rains in Senegal. The rainy season across the Sahel was characterized by enhanced rainfall activity, with amounts approximately 15% above the long term climatological mean for the period 1950-2005, a 60% increase in rainfall, with respect to the most recent 30 year climatological mean for 1971-2000 [37].

### Conclusions

Since the beginning of the seventh cholera pandemic in Africa in 1970, the disease remains an ongoing cause of morbidity and mortality. The persistence of cholera in many African countries raises a legitimate question whether cholera is and has always been endemic in Africa [33]. In any case, cholera, a model for climate-related infectious diseases, can be concluded to be driven by climate in Africa [8], [9], [10], [11], as in Asia and Latin-America [4], [5], [38].

In conclusion, results of the analysis of cholera and rainfall dynamics for Dakar, Senegal, during 2005 show rainfall pattern that generated the floods in Dakar that occurred during August, 2005, concurrently with a significant increase in the number of cholera cases. The influence on cholera transmission of this intense precipitation over a densely populated and crowded region was detectable for both Dakar and Thiès, Senegal. An objective of this study was to develop a predictive framework, integrating knowledge derived from theoretical and empirical models of cholera [7], [39], [40] with high resolution rainfall forecasts at subseasonal time scales to be able to initiate timely epidemic preparedness. Senegal and West Africa will certainly be subject to future extreme weather events, including floods associated with climate change [41], [42], [43] since the tropical Atlantic experiences regime shifts and ocean warming attributable to anthropogenic activities. Therefore, attention must be paid to both natural and human induced environmental factors if appropriate action is to be taken to prevent cholera and other disease epidemics in the region [15], [20].

## Supporting Information

Figure S1 **Daily rainfall country mean annually accumulated anomaly between 2002 and 2005.** Mean is represented by the black line, the grey surface represents the (+/−) standard deviation around the mean.
(TIF)

Figure S2 **Cross-correlation diagram between cholera cases time-series and rainfall for Dakar between May 10 and December 31, 2005.** Lag time unit is days.
(TIF)

Table S1 **Annual cholera morbidity in Senegal and its frontline countries from 1970 to 2010 (source WHO)**
(DOC)
