# Supplementary figures and images for: Cholera Outbreak in Senegal in 2005: Was Climate a Factor?
Source: PLoS One. 2012 Aug 31;7(8):e44577. doi: 10.1371/journal.pone.0044577 (PMC3432123; doi:10.1371/journal.pone.0044577)

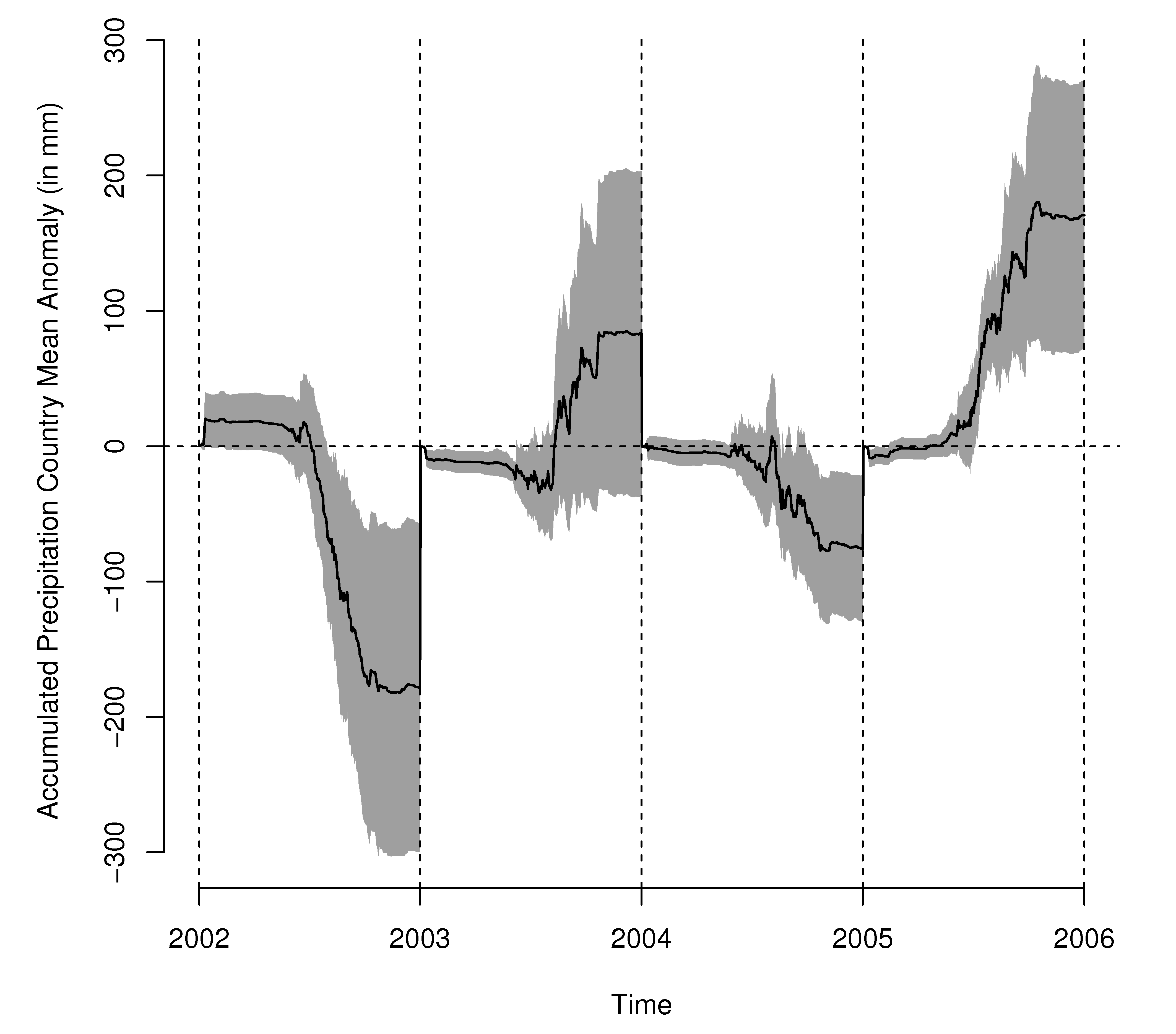

Supplement: Figure S1 — Daily rainfall country mean annually accumulated anomaly between 2002 and 2005. Mean is represented by the black line, the grey surface represents the (+/−) standard deviation around the mean. (TIF) [file pone.0044577.s001.tif]

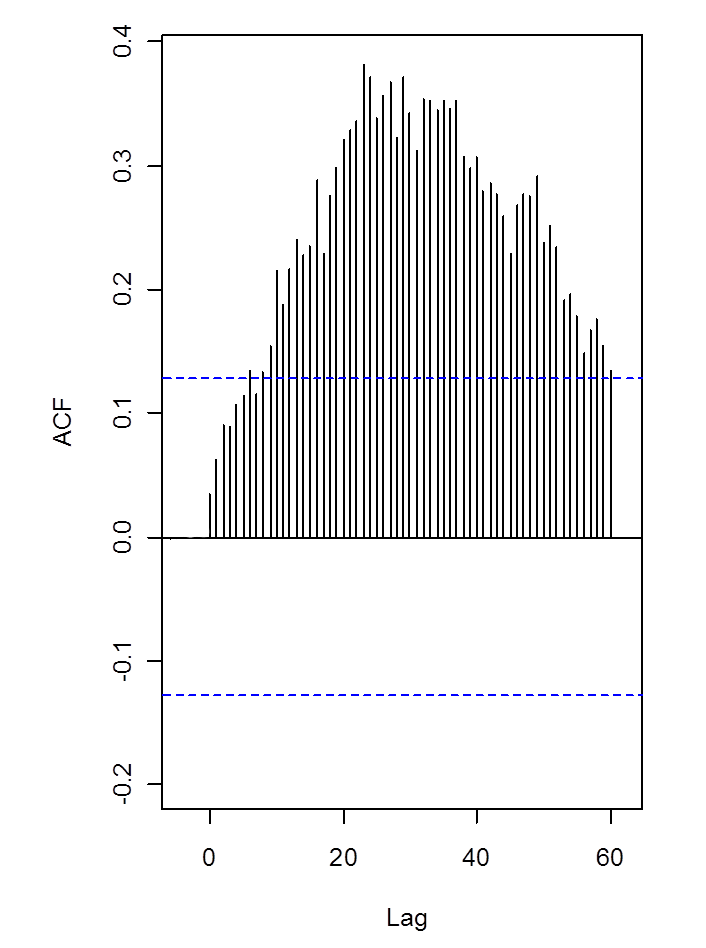

Supplement: Figure S2 — Cross-correlation diagram between cholera cases time-series and rainfall for Dakar between May 10 and December 31, 2005. Lag time unit is days. (TIF) [file pone.0044577.s002.tif]
