# Supplementary material for: Cholera Outbreak in Senegal in 2005: Was Climate a Factor?
Source: PLoS One. 2012 Aug 31;7(8):e44577. doi: 10.1371/journal.pone.0044577 (PMC3432123; doi:10.1371/journal.pone.0044577)
Supplement: Table S1 — Annual cholera morbidity in Senegal and its frontline countries from 1970 to 2010 (source WHO) (DOC) [file pone.0044577.s003.doc]

**Cholera outbreak in Senegal in 2005: was climate a factor?**

Guillaume Constantin de Magny, Wassila Thiaw, Vadlamani Kumar, Noël M. Manga, Bernard M. Diop, Lamine Gueye, Mamina Kamara, Benjamin Roche, Raghu Murtugudde, Rita R. Colwell.

Table S 1. Annual cholera morbidity in Senegal and its frontline countries from 1970 to 2010 (source WHO).

|  | Senegal | Gambia | Guinea | Guinea-Bissau | Mali | Mauritania |
| --- | --- | --- | --- | --- | --- | --- |
| 2010 | 3 |  |  |  |  |  |
| 2009 | 4 |  | 42 | 5 |  |  |
| 2008 | 1,283 | 1 | 513 | 14,323 | 153 |  |
| 2007 | 3,984 | 12 | 8,546 | 153 |  | 3 |
| 2006 | 365 | 0 | 3,242 | 37 | 7 | 25 |
| 2005 | 31,719 | 214 | 3,821 | 25,111 | 1,178 | 4,132 |
| 2004 | 1,227 |  | 1,516 | 155 | 2,839 |  |
| 2003 |  |  | 6 | 290 | 1,455 | 34 |
| 2002 |  |  | 61 | 842 | 18 | 80 |
| 2001 |  |  | 392 |  | 67 |  |
| 2000 |  |  | 519 |  | 1,885 |  |
| 1999 |  |  | 599 |  | 6 |  |
| 1998 |  |  | 881 | 126 |  |  |
| 1997 | 371 |  |  | 20,555 | 6 | 462 |
| 1996 | 16,107 | 7 | 287 | 8,397 | 5,723 | 4,534 |
| 1995 | 3,332 | 15 | 6,506 | 119 | 2,191 |  |
| 1994 |  | 1 | 31,415 | 15,296 |  |  |
| 1993 |  |  |  |  |  |  |
| 1992 |  |  |  |  |  |  |
| 1991 |  |  |  |  |  |  |
| 1990 |  |  |  |  |  |  |
| 1989 |  |  |  |  |  | 700 |
| 1988 | 390 |  |  |  |  | 575 |
| 1987 | 3,150 |  |  | 6,000 | 352 | 1,578 |
| 1986 | 476 |  | 286 | 200 | 1,916 | 3,734 |
| 1985 | 2,988 | 2 |  |  | 3,759 | 259 |
| 1984 | 728 |  |  |  | 1,795 | 166 |
| 1983 |  |  |  |  |  |  |
| 1982 |  |  |  |  |  |  |
| 1981 | 428 |  |  |  |  |  |
| 1980 |  |  |  |  |  |  |
| 1979 | 103 |  |  |  |  |  |
| 1978 | 315 |  |  |  |  |  |
| 1977 |  |  |  |  |  |  |
| 1976 |  |  |  |  |  |  |
| 1975 |  |  |  |  |  |  |
| 1974 |  |  |  |  | 130 |  |
| 1973 |  |  |  |  | 219 | 150 |
| 1972 | 379 |  |  |  | 2 | 148 |
| 1971 | 265 |  |  |  | 4,792 | 1,139 |
| 1970 |  |  | 2,000 |  | 2,665 |  |
| Total | 67,617 | 252 | 60,632 | 91,609 | 31,158 | 17,719 |
| Mean | 3,380.85 | 31.50 | 3,566.59 | 6,107.27 | 1,483.71 | 1,107.44 |
